# Hydroxyapatite deposits of the hand and wrist: a diagnosis not to be ignored

**DOI:** 10.11604/pamj.2021.38.408.29253

**Published:** 2021-04-29

**Authors:** Daniel Bernier, Emilie Marteau, Steven Roulet, Hèdi Antar, Ameur Triki, Jacky Laulan, Guillaume Bacle

**Affiliations:** 1 Department of Orthopedic Surgery, Hand Surgery Unit, Trousseau University Hospital, Medical University François Rabelais of Tours, Tours, France,; 2 Department of Orthopedic Trauma and Burns Center Ben Arous, Tunis, Tunisia,; 3 Mohamed Kassab Institute of Orthopedics, La Manouba, Tunis, Tunisia,; 4 Unité Mixte de Recherche, Institut National de la Santé et de la Recherche Médicale (INSERM), iBrain, Medical University François Rabelais of Tours, Tours, France

**Keywords:** Anti-inflammatory drugs, arthritis, hand, hydroxyapatite, wrist

## Abstract

Hydroxyapatite crystal deposition disease (HADD) of the hand and wrist is rare but can cause acute inflammatory syndromes that mimic infectious arthritis. These symptoms, which rapidly resolve with systemic anti-inflammatory drugs, are a source of diagnostic errors and inappropriate treatment. It is of crucial importance to make the diagnosis in order to avoid iatrogenic surgical management. The aim of this study was to determine the clinical and radiographic signs and the key features on which diagnosis depends. Treatment effectiveness and the course of the disease were also examined. Between 1992 and 2008, 12 patients consulted for an isolated acute local inflammatory syndrome of the hand or wrist, which was accompanied by a unique radiographic picture of calcific density. All patients were reassessed clinically and radiographically with a minimum follow-up of 2 years. All patients had presented with acute local inflammatory syndromes. Nine patients had edema and 8 had swelling and erythema. No patient had fever. The course was favorable in 11 patients and one patient required surgery. No patient had a recurrence at the mean final follow-up of 90 ± 64 months. The symptoms associated with hydroxyapatite crystal deposits suggest septic arthritis with acute joint inflammation. The radiological appearance is characteristic and corrects the diagnosis. Oral anti-inflammatory treatment gives more rapid spontaneous improvement, with complete and long-lasting resolution.

## Introduction

The acute manifestations of hydroxyapatite crystal deposition disease (HADD) are rare and little known. They are a source of diagnostic errors and iatrogenic management [1-5]. In the hand and wrist the clinical presentation is often highly inflammatory, suggesting acute arthritis, and may mimic an infectious cause. In the wrist, septic arthritis is rare and accounts for less than 5% of cases of acute inflammatory arthritis [6]. Most cases of septic arthritis are due to microcrystals, generally related to gout or articular chondrocalcinosis [6-8]. In the fingers, an infectious cause is more frequent but a metabolic origin is possible, in particular gout or more rarely HADD [2,8]. Although an infectious cause is in principle the first to be considered, the possibility of HADD must be suggested if there is a clinical picture of extremely painful inflammation of rapid onset, in particular in the absence of a portal of entry and a context of decreased immune defenses [5,9]. The diagnosis of HADD may reasonably be made based solely on the presence of calcifications whose radiographic appearance is characteristic [1,10,11]. The course is spontaneously favorable but the cure may be hastened by 2-week treatment with systemic nonsteroidal anti-inflammatory drugs (NSAIDs) [3,12]. Surgery is only indicated if symptoms persist beyond the acute episode [11]. Conversely, treatment for septic arthritis is undoubtedly surgical and NSAIDs are formally contra-indicated [6,9]. The aim of the study was to determine the clinical and radiographic signs of HADD of the hand or wrist and the key features that establish the diagnosis. The effectiveness of treatment and the course of the disease were also studied.

## Methods

**Ethical approval and patient inclusion:** this single-center descriptive study received prior approval from the ethics committee of our institution. From January 1992 to February 2018, patients who consulted in our department for acute inflammation related to HADD of the hand or wrist were included retrospectively. Inclusion criteria were i) an isolated acute painful inflammatory episode of the hand or wrist, associated with ii) a unique radiographic picture of calcific density located adjacent to a joint or tendon insertion of the painful area. Exclusion criteria were i) microcrystal-related metabolic disorder (gout or joint chondrocalcinosis), ii) chronic renal failure, iii) autoimmune disease such as connectivitis (systemic scleroderma/CREST syndrome, dermatomyositis), and iv) a history of intra-articular injection, surgery or significant injury of the area involved.

**Clinical and radiographic data collection at inclusion and at follow-up:** the data collected for each patient were age at time of diagnosis, sex, dominant side, characteristics and precise location of symptoms, and radiographic findings. The local and general signs sought during the initial episode were fever, pain, erythema, edema, swelling and functional disability. The initial treatment (nonsteroidal anti-inflammatory drugs (NSAIDs), corticosteroids, joint infiltrations and/or surgery) and its effectiveness were noted. All patients included were seen again in consultation for clinical and radiographical evaluation by an independent examiner. During the last follow-up visit, the occurrence of a new acute episode that could indicate a possible recurrence was systematically sought. The patient was also questioned about time to resolution of the initial symptoms and persistence of any symptoms. On the initial radiographs and on those obtained at the last consultation, the location of images of calcific density, their appearance (homogeneous or fragmented) and the presence of any other calcifications were noted.

## Results

**Figure 1:**
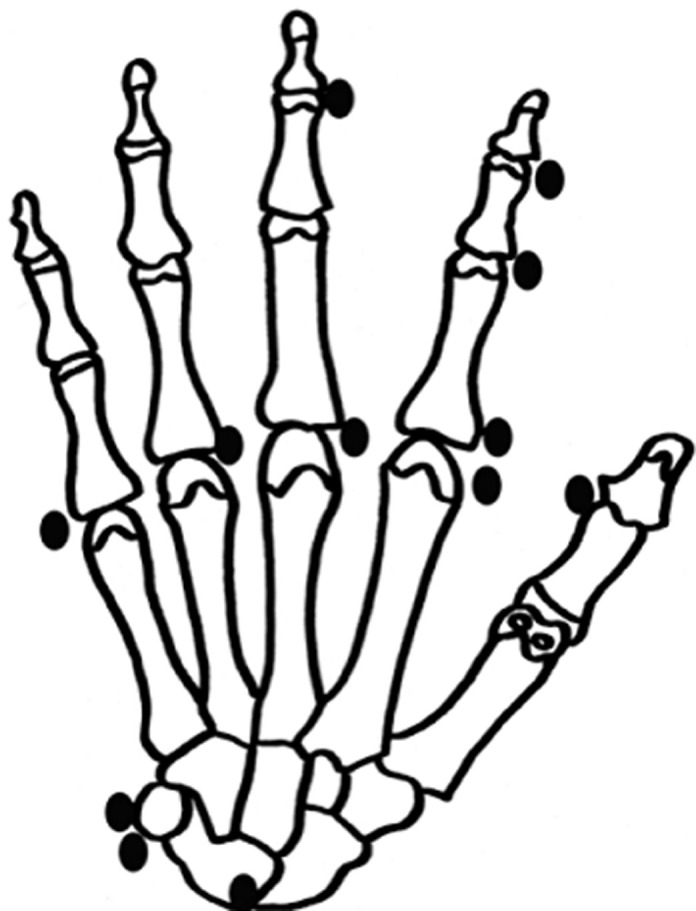
representation of locations of hydroxyapatite crystal deposits in the patient series

**Figure 2:**
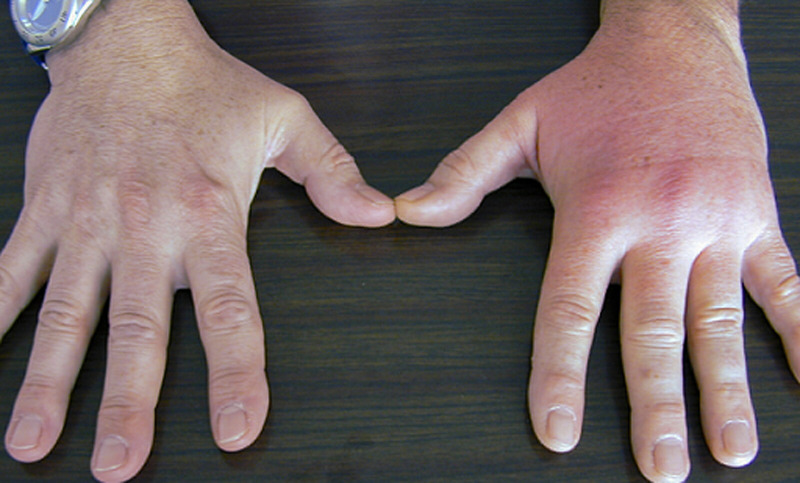
local inflammatory syndrome centered on the metacarpophalangeal (MCP) joint of the third ray, concurrent with resorption of a hydroxyapatite deposit

**Figure 3:**
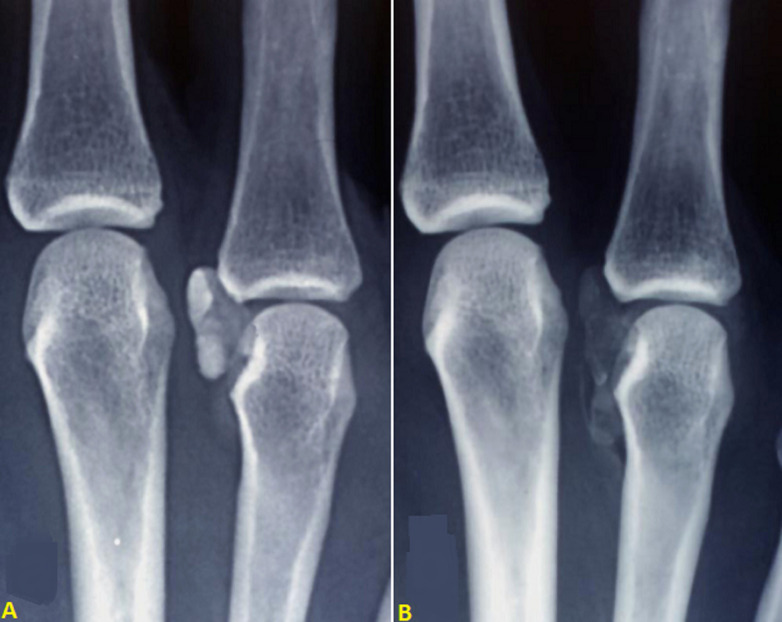
anteroposterior radiograph centered on the inflammatory joint; A) radiographic appearance in the acute phase (beginning of resorption); note the clear outline and periarticular location of the calcification; B) appearance 3 weeks later; gradual disappearance with spread of the calcification and attenuation of its calcific density

Twelve patients were included, 5 men and 7 women, whose mean age was 56 ± 14 years (range, 35 to 78 years). Their characteristics are presented in the (Table 1) and the locations of hydroxyapatite deposits are shown in (Figure 1). Two patients who had died at the time of the last clinical review were considered as lost to follow-up (cases 4 and 7). Ten patients were seen at the final follow-up visit. Mean duration of follow-up was 90 ± 64 months. In all 12 patients, the clinical manifestations during the acute episode were pain and major functional disability. Significant local edema was present in 9 cases and erythema and swelling in 8 cases (Figure 2). No patients had fever during the acute phase. The diagnosis of HADD was confirmed by radiography in all patients. Radiographs were obtained at the beginning of the acute episode in 10 patients and always showed a homogeneous deposit (Figure 3). In two other patients (cases 10 and 11), radiographs were obtained 2 weeks after symptom onset and showed heterogeneous calcifications undergoing resorption in the painful area. The deposits were located near the metacarpophalangeal joints in 5 cases (Figure 3), the interphalangeal joints in 4 cases, at the insertion of the flexor carpi ulnaris tendon on the pisiform in 2 cases, and in the carpal canal at the radiocarpal joint in 1 case. No patient was primarily treated surgically. Eleven patients were prescribed medical treatment with anti-inflammatory drugs. The patient who received no treatment (case 5) had complete resolution of pain, which together with functional disability was the main symptom in the acute episode. The course was favorable in 11 patients (92%) with complete resolution of symptoms in 2 to 3 weeks. One patient (case 6) still had residual calcifications in the radial collateral ligament of the index finger after the initial acute episode. Intra-articular injection with cortisone derivatives gave only temporary improvement. Because of persistent pain, the residual calcific deposits were surgically excised 3 years after the acute episode and the course was subsequently favorable. At last follow-up, no patient had had a new acute inflammatory episode and no new calcific deposits were observed on the final radiographs.

**Table 1:** clinical data and follow-up of the study patients

Patient	Age at diagnosis (years)	Sex	Dominant/ Involved side	Location	Clinical features	Appearance of hydroxyapatite deposits	Treatment	Time to symptom resolution (weeks)	Follow-up (months)
1	47	F	R/L	DIP	pain, edema, erythema, swelling, disability	homogeneous	NSAIDs	4	95
2	64	F	R/R	MCP	pain, edema, erythema, swelling, disability	homogeneous	NSAIDs	5	88
3	78	F	L/R	MCP	pain, edema, erythema, swelling, disability	homogeneous	NSAIDs	3	54
4	65	M	R/R	Pisiform	pain, edema, erythema, swelling, disability	homogeneous	NSAIDs	—	—
5	71	M	R/R	Pisiform	pain, disability	homogeneous	none	8	26
6	35	F	R/R	PIP	pain, swelling, disability	homogeneous	corticosteroids	persistent pain	56
7	52	M	R/L	MCP	pain, edema, erythema, swelling, disability	homogeneous	NSAIDs	—	—
8	45	F	R/R	MCP	pain, edema, swelling, disability	homogeneous	corticosteroids	4	221
9	55	M	R/L	Carpal tunnel	pain, edema, disability	homogeneous	NSAIDs	6	97
10	54	F	R/L	MCP	pain, edema, erythema, disability	heterogeneous	NSAIDs	3	159
11	39	M	R/R	PIP	pain, erythema, swelling, disability	heterogeneous	NSAIDs	4	24
12	72	F	R/R	DIP	pain, edema, disability	homogeneous	NSAIDs	5	27

F: female; M: male; R: right; L: left; DIP: distal interphalangeal joint; PIP: proximal interphalangeal joint; MCP: metacarpophalangeal joint; NSAIDs: nonsteroidal anti-inflammatory drugs; —: patient deceased.

## Discussion

Hydroxyapatite crystal deposition disease may be asymptomatic or may simply cause swelling [4]. The onset of an acute inflammatory episode is generally concurrent with resorption of the calcified deposit, as shown by the follow-up of the patients in our series and the data of the literature [2,3,5,13]. The clinical manifestations in the acute phase are often impressive, with acute juxta-articular inflammation suggesting septic arthritis [1,5]. Pain is intense and is present in all cases in the literature [1-3,10,11,14]. In our patient series functional disability was always present, with edema in three-quarters of cases and objective signs of inflammation in two-thirds. However, no patient had fever. The mean age of the patients in our study was 56 years, with a range of 35 to 78 years. This corresponds to the findings of the literature for sites involving the hand, where the mean age was 50 years [1,10-12,15]. In their study, Kim *et al*. [14]. Found a significantly higher mean age in the group with peritendinitis (45 years) than in the group with periarthritis (35 years). HADD is exceptional in children [16]. The youngest patients with HADD of the hand and wrist were aged 8 and 9 years [4,16]. We found a female predominance with a sex ratio of 1.4. This difference is even more marked in the literature with a sex ratio ranging from 2 to 9 [10,11,14]. The first location of HADD to be described was the insertion of *flexor carpi ulnaris* on the pisiform [17]. This is the most frequent location in the wrist and was found in 2 of our 3 cases [5,10,11]. More rarely, locations at the tendons of *extensor carpi ulnaris, flexor carpi radialis, flexor pollicis longus and abductor pollicis longus* have also been reported [2,4,14]. The third case (case 9) presented with acute carpal canal syndrome during the inflammatory flare-up related to HADD located at the radiocarpal joint. The course was favorable under medical treatment with disappearance of the calcification and complete resolution of the neurological symptoms. Even in this particular location, first-line medical treatment remains indicated, but if there is no resolution, surgical treatment with opening of the carpal annular ligament and excision of the calcifications appears justified [12,15,17].

As in the series of Kim *et al*. [14] the hand was involved in the majority of our cases. In the literature, periarticular involvement predominated in the MCP. Moyer *et al*. [10] observed no involvement of the interphalangeal joints, while Kim and Park found 18% of proximal interphalangeal (PIP) joint involvement and 12% of distal interphalangeal (DIP) joint involvement [14]. However, in our series, locations at the interphalangeal joints were almost as frequent, accounting for 4 of the 9 cases involving the hand. The diagnosis was made on radiographs in all patients. The appearance of HADD on radiographs obtained at the beginning of the acute episode is typical, showing a unique image of calcification, relatively homogeneous, rounded or plurilobulated, amorphous (with no cortical or cancellous bone), located at a joint or a tendon insertion [1,2,10-12,14]. Generally, during the acute episode, the calcification breaks up and becomes poorly outlined, with an indistinct margin, less dense and with a flocculent or cloudy appearance; it disappears completely or almost completely within 3 months [2]. The clinical course was favorable and the symptoms completely resolved in all patients except one, who continued to experience pain after resolution of the initial inflammatory flare. Moyer *et al*. [10], like Dilley and Tonkin [2], observed a favorable course with medical treatment in all their patients. It is probably desirable to avoid corticosteroid injections, as these may cause HADD and increase the possibility of recurrence [12,18,19].

All the patients in our study received medical treatment except one patient who was not diagnosed initially and who recovered spontaneously without sequelae. In the literature, the reference first-line treatment is medical, with anti-inflammatory agents [1-3,10]. The symptoms resolve from within a few days to 3 or 4 weeks [1,2,5]. In 2018, *Kim et al*. [11] evaluated treatment effectiveness and concluded that if the pain did not resolve after 6 months of anti-inflammatory treatment, surgical management should be proposed. In their study, 33% of patients were eligible for surgical treatment, but 50% had multiple locations in the hand and wrist. This multifocal involvement does not correspond to the classic presentation of idiopathic HADD and may explain the proportion of their patients who did not respond to medical treatment. Among our patients, who all had a single location of HADD, only one patient still experienced pain after 6 months of medical treatment, with incomplete resorption of the calcific deposit, and required surgical treatment. None of our patients had a recurrence. Most authors report long-lasting resolution of symptoms and the absence of recurrence [1,2,10]. However, Kim *et al*. [14] observed 31% of recurrence for peritendinous locations but none for periarticular locations. It should be noted that 3 of their 4 cases of recurrence were initially treated with corticosteroid injections. Our single case with persistent symptoms was a periarticular and not a tendinous location.

The symptoms in the acute phase are similar to those of infectious arthritis. However, fever is not a discriminant criterion, as it may be absent in infectious arthritis whereas moderate fever may accompany an inflammatory flare in HADD [5,6,9,13]. Similarly, laboratory findings are not systematically abnormal in septic arthritis of the hand and wrist whereas they may sometimes be slightly abnormal in HADD [5]. The diagnosis of an inflammatory flare associated with HADD of the hand or wrist is thus based on the presence of characteristic calcifications on radiographs of the affected area [3,12]. Because of their typical appearance and the absence of associated abnormalities, they are easily distinguished from the calcifications observed in joint chondrocalcinosis and gout [3,15]. In order to reveal these calcific deposits, which may be superimposed over bone structures, anteroposterior, lateral and oblique views must systematically be obtained. If the patient is seen 1 to 2 weeks after the onset of the acute episode, the calcific deposit has been partly resorbed and has a heterogeneous appearance, but at this stage the symptoms are already resolving, as was the case in 2 of our patients who were seen late [14].

## Conclusion

The calcific deposits of HADD can cause intense inflammation of acute onset that may wrongly suggest septic arthritis. The signs, symptoms and laboratory findings do not allow a definitive diagnosis, whereas the treatment of these two entities is radically opposite. When confronted by an acute inflammatory picture of infectious appearance of the hand or wrist, it is indispensable to obtain radiographs with several different views of the affected area before starting any treatment. The presence of a typical calcified deposit is sufficient in itself to establish the diagnosis of HADD and avoid unnecessary surgery for the patient.

### What is known about this topic

- The clinical manifestations of hydroxyapatite crystal deposition disease in the acute phase are often impressive, with acute juxta-articular inflammation, pain is intense that may wrongly suggest septic arthritis;
- The appearance of HADD on radiographs obtained at the beginning of the acute episode is typical, showing a unique image of calcification, relatively homogeneous, rounded or plurilobulated, amorphous (with no cortical or cancellous bone), located at a joint or a tendon insertion;
- The reference first-line treatment is medical, with anti-inflammatory agents. The symptoms resolve from within a few days to 3 or 4 weeks.

### What this study adds

- The signs, symptoms and laboratory findings do not allow a definitive diagnosis, whereas the treatment of these two entities is radically opposite;
- Locations of HADD at the interphalangeal joints is almost as frequent than at the metacarpophalangeal joint;
- The presence of a typical calcified deposit is sufficient in itself to establish the diagnosis of HADD and avoid unnecessary surgery for the patient.
